# Drinking in Summer

**Published:** 1871-06

**Authors:** 


### DRINKING IN SUMMER.

THE longer a person can put off drinking a glass of water on a hot summer's day, the better it will be for him; for if he drinks largely early in the day, the thirst will be increased, with an uncomfortable sense of fullness, large perspiration, increased liability to colds, with a delibitated condition of the system.

In taking a glass of water, or other cold drink, it is better to take but a single swallow at a time, removing the glass from the lips for a few seconds; thus the thirst will be quenched with half the amount of water, and danger is avoided of sudden prostration. Half a dozen swallows thus taken will quench the thirst more completely than twice the amount if taken continuously without removing the glass from the lips.

If a person is very thirsty, chewing lumps of ice is safer, better, and more effective, than five times the amount in the form of cold water. If very warm, it is safer to hold the glass for a minute or two encircled with the fingers and palm of the hand. This cools the blood a little, and at the same time tempers the water.

